# Novel Sustainable Composites Incorporating a Biobased Thermoplastic Matrix and Recycled Aerospace Prepreg Waste: Development and Characterization

**DOI:** 10.3390/polym15163447

**Published:** 2023-08-18

**Authors:** José Antonio Butenegro, Mohsen Bahrami, Yentl Swolfs, Jan Ivens, Miguel Ángel Martínez, Juana Abenojar

**Affiliations:** 1 Materials Science and Engineering and Chemical Engineering Department, IAAB, University Carlos III Madrid, 28911 Leganés, Spain; 2 Department of Materials Engineering, KU Leuven, Kasteelpark Arenberg 44, B-3001 Leuven, Belgium; 3 Mechanical Engineering Department, Universidad Pontificia Comillas, Alberto Aguilera 25, 28015 Madrid, Spain

**Keywords:** polymer matrix composites (PMCs), carbon fiber-reinforced polymers, discontinuous reinforcement, thermoplastic polymers, mechanical properties

## Abstract

Carbon fiber-reinforced polymer (CFRP) composite materials are widely used in engineering applications, but their production generates a significant amount of waste. This paper aims to explore the potential of incorporating mechanically recycled aerospace prepreg waste in thermoplastic composite materials to reduce the environmental impact of composite material production and promote the use of recycled materials. The composite material developed in this study incorporates a bio−based thermoplastic polymer, polyamide 11 (PA11), as the matrix material and recycled aerospace prepreg waste quasi-one-dimensionally arranged as reinforcement. Mechanical, thermal, and thermomechanical characterizations were performed through tensile, flexural, and impact tests, as well as differential scanning calorimetry (DSC) and dynamic mechanical analysis (DMA). Compared to previous studies that used a different recycled CFRP in the shape of rods, the results show that the recycled prepregs are a suitable reinforcement, enhancing the reinforcement-matrix adhesion and leading to higher mechanical properties. The tensile results were evaluated by SEM, and the impact tests were evaluated by CT scans. The results demonstrate the potential of incorporating recycled aerospace prepreg waste in thermoplastic composite materials to produce high-performance and sustainable components in the aerospace and automotive industries.

## 1. Introduction

Carbon fiber-reinforced polymer (CFRP) composite materials play a crucial role in various engineering applications due to their exceptional mechanical properties and versatility. CFRPs have revolutionized several industries, including aerospace, automotive, construction, marine, and sports equipment manufacturing [1]. In the aerospace and automotive industries, CFRPs enable lighter and more fuel-efficient and innovative designs while improving crashworthiness in the case of automotive [2,3,4,5]. CFRPs benefit sports equipment, marine vessels, and construction through lighter, stiffer, and more durable products, improved performance, and longevity [6,7,8,9]. CFRPs possess a high strength-to-weight ratio, corrosion resistance, thermal stability, and fatigue resistance. These inherent properties facilitate the development of advanced engineering solutions, foster innovation, and drive significant technological advancements. However, the traditional production processes of CFRPs involve significant waste generation, primarily due to the use of carbon fibers and thermoset matrices and the difficulty of recycling them efficiently. The accumulation of composite waste poses environmental challenges and raises concerns regarding the sustainable use of resources [10,11]. Therefore, there is a pressing need to explore alternative approaches that minimize waste and promote the use of sustainable materials in composite manufacturing.

Over the last few decades, there has been a notable surge in the adoption of thermoplastic composites, driven by various industries recognizing their immense potential. While the automotive sector has been at the forefront, leveraging the advantages of reduced cycle times, other markets, including the aerospace industry, are increasingly acknowledging the numerous benefits offered by thermoplastic composites [12]. Advantages of thermoplastic matrices over thermoset matrices include shorter processing times since no chemical reactions need to be completed, higher toughness leading to improved impact resistance, and easier recyclability. However, thermoplastic matrices have some drawbacks compared to thermoset matrices, such as more complex flow behavior due to having longer chains, significant shrinkage upon cool-down, and difficulties in achieving good adhesion with fibers due to their often less polar nature [13,14].

PA11 is a particularly interesting polymer, as it is a bio-based polyamide derived from castor oil, offering a reduced carbon footprint compared to oil-based polymers [15]. PA11 is gaining significant traction in various industries due to its low moisture absorption, impact, fatigue, chemical and aging resistance, and dimensional stability [16]. PA11 exhibits a relatively low melting temperature compared to other polyamides, such as PA6 and PA66, resulting in reduced energy consumption during manufacturing processes [17].

In general, all industries that use composite materials have a high degree of waste. In particular, the aerospace industry, which has specific requirements of lightweight, high strength, and exceptional performance in extreme conditions, generates a substantial volume of composite waste throughout the production process and at the end-of-life of aircraft components. The waste includes scraps, trimmings, and offcuts that result from the manufacturing processes. Moreover, retired or damaged components that are no longer suitable for flight contribute to the overall volume of discarded materials.

Motivations for recycling composite materials include cost-effectiveness, circular economy principles, and environmental sustainability [18,19]. Recycling offers a cost-effective solution by obtaining valuable materials from waste streams, reducing production costs. Embracing a circular economy model minimizes resource extraction, promotes waste reduction, and enhances long-term sustainability. The recycling of composites contributes to environmental sustainability by diverting waste from landfills, conserving energy, and reducing greenhouse gas emissions. These motivations drive the exploration of alternative approaches to minimize waste and promote sustainable materials in composite manufacturing.

Thermal, chemical, and mechanical recycling are the three main approaches commonly employed in the recycling of composites [20]. Each method has its advantages and limitations. Thermal recycling involves subjecting composites to high temperatures to break them down but requires substantial energy inputs [21,22]. Chemical recycling involves the degradation of the polymer matrix to recover the fibers, but scalability and the handling of chemicals pose challenges [23,24]. Mechanical recycling involves physically shredding and grinding composites and stands out among the routes for composite recycling due to its ability to address multiple challenges [25]. Unlike thermal and chemical recycling, mechanical recycling avoids high energy requirements and scalability issues while at the same time reducing the emission of pollutant gases and the problems associated with the disposal of chemicals and solvents. A significant objective of mechanical recycling is to preserve the maximum performance of the recycled materials, ensuring their suitability for high-value applications [26,27].

Some authors use milling for mechanically recycling CFRPs, but drawbacks arise, including lost value for long fiber CFRPs due to shorter lengths, reduced reinforcement, and potential fiber damage, impacting strength [28]. Most authors usually focus on methods that involve removing the previous matrix to enable the recovery and reuse of carbon fibers. Gillet et al. [29] emphasized the positive impact of longer fiber lengths on the strength of composites, highlighted the need for improved fiber alignment to reduce scatter and achieve desired properties, and recommended fiber/matrix separation techniques for recovering healthy fibers in the production of high-strength recycled composites. Barnett et al. [30] investigated recycled carbon fiber composites for automotive crash management, utilizing pyrolysis as a recycling method. Their study revealed the high specific energy absorption at dynamic rates and excellent crash performance, offering a cost-effective and ecofriendly alternative to continuous virgin fiber laminates while maintaining crashworthiness.

To the best of the authors’ knowledge, only a few studies have attempted to mechanically recycle CFRPs without removing the prior matrix or milling the CFRP. Stelzer et al. [31] developed a cost-efficient, large-scale, one-shot compression molding process for structural automotive component manufacturing. Multilayered hybrid carbon fiber materials, including recycled fibers, were utilized. Unidirectional prepreg tape was chopped into rectangular platelets (approx. 50 mm × 8 mm) and randomly distributed, maintaining manufacturability and mechanical performance, with potential for circularity in hybrid carbon fiber composites. Wan and Takahashi [27] employed compression molding to create ultrathin chopped carbon fiber tape composites with polyamide 6 as the matrix. The composites, featuring reinforcement tape lengths of 6 to 24 mm, exhibited tensile and compressive modulus values nearly independent of the reinforcement length. However, increasing tape length slightly improved structural integrity, enhancing both tensile and compressive strength.

This paper aims to develop and characterize a novel composite material that incorporates mechanically recycled expired aerospace prepreg waste as reinforcement in a thermoplastic matrix. Specifically, polyamide 11 (PA11), a biobased polyamide, is used as the matrix material, and a quasi-one-dimensional reinforcement arrangement is pursued. Compared to previous studies, the objective is to enhance the reinforcement-matrix adhesion, improving the overall mechanical and thermomechanical properties of the composite materials. This research revolves around recycling and circular economy, exploring the potential of incorporating mechanically recycled aerospace prepreg waste in thermoplastic composite materials.

## 2. Materials and Methods

### 2.1. Materials

The polyamide 11 (PA11) employed was supplied by Arkema (Arkema, Barcelona, Spain) in the form of pellets, which were carefully stored to avoid exposure to direct sunlight. PA11 was chosen as the matrix material due to being biobased and to its relatively low melting point (T_m_), which results in lower energy consumption during processing, as well as its exceptional mechanical properties, including high tensile strength, impact and fatigue resistance, chemical and aging resistance, and low moisture absorption [26,32].

The reinforcement consists of an expired aeronautical prepreg, with the trade name HexPly M21EV/IMA, supplied by Hexcel (Hexcel Composites, Madrid, Spain). The prepreg is composed of an epoxy resin and carbon fibers. It uses a high-performance, high-strength epoxy matrix for use in primary aerospace structures, with a density of 1.28 g/cm^3^ and a glass transition temperature of 180 °C. The prepreg presents a longitudinal and transverse modulus of 178 GPa and 11.8 GPa, respectively, a longitudinal Poisson’s ratio of 0.39, and an in-plane shear modulus of 5.2 GPa [33]. Prior to being used as reinforcement, the prepreg was mechanically reduced to a length of 40 ± 2 mm and a width of 1.2 ± 0.2 mm, with a thickness of 200 ± 10 μm.

### 2.2. Composite Manufacturing

**Figure 1:**
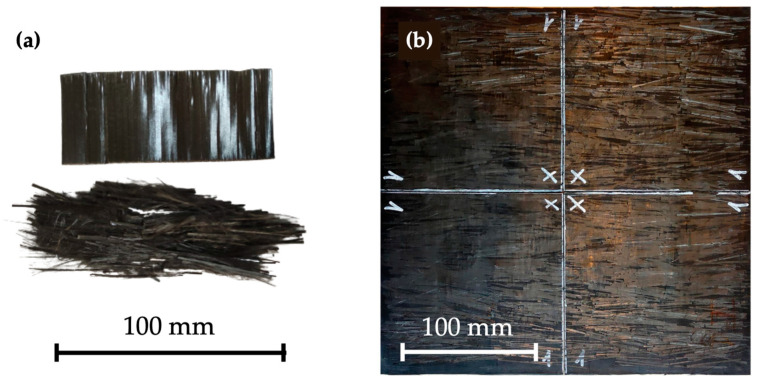
(**a**) Detail of a section of the prepreg (up) and of the mechanically recycled prepreg (down). (**b**) Manufactured composite sheet for impact testing.

The composite manufacturing process is carried out in a Fontijne Press TPB374 (Fontijne Press, Barendrecht, The Netherlands) hydraulic press and involves two stages. In the first stage, PA11 pellets are used to manufacture PA11 sheets of 0.6 ± 0.1 mm between aluminum plates of 280 × 280 mm^2^. In the second stage, the reinforcement is placed above and below the PA11 sheet following a one-dimensional arrangement, and the compaction process is performed again. The specimens used for tensile, flexural, and thermomechanical tests contained reinforcement positioned in one direction only, while the reinforcements for impact tests were stacked in 0/90/90/0 configuration. Figure 1 presents a detail of the mechanically recycled prepreg alongside a composite sheet manufactured for impact testing.

For the compaction process, the cycle used was optimized by Bahrami et al. [17]. Pressure and temperature were increased in a stepwise and linear manner, respectively, during the first ten minutes until a pressure of 0.5 MPa and a temperature of 200 °C were reached, slightly more than 10 °C above the T_m_ of PA11. Subsequently, the pressure was maintained at 0.5 MPa throughout the pressing process until the release of the plate. The temperature was initially maintained at 200 °C for 10 min and then decreased in two linear steps. The first step consisted of cooling with compressed air, had a duration of 30 min and a slope of −2 °C/min so that a temperature of 140 °C was reached at the end of the step. During this step, the cooling rate was intentionally slower than the rest of the cycle. This deliberate approach allowed for a gradual surpassing of the end of the crystallization peak determined by DSC. Additionally, a slower cooling rate gives more time for the polymer chains to arrange themselves in a more ordered manner, leading to larger and better-defined crystalline regions and, therefore, an enhanced performance. The second step included cooling with compressed air and water, which had a duration of 20 min and a slope between −5 and −6 °C/min.

### 2.3. Mechanical Testing

#### 2.3.1. Tensile Testing

Tensile tests were conducted in accordance with ISO 527-5 using a ZwickRoell Z100 (Zwick, Ulm, Germany) universal testing machine equipped with a 100 kN load cell. The specimens were securely clamped using hydraulic grips with a pressure of 100 bar, and the distance between the grips was 150 mm. To prevent slippage during testing, P180 sandpaper was inserted between the specimens and the grips. The tests were carried out under controlled humidity and temperature conditions (23 °C and 50% RH). The longitudinal strain of the specimens was measured by means of a videoXtens 3-300 video-extensometer system (Zwick, Ulm, Germany), allowing for accurate and precise measurements of deformation during testing. Full-field strain mapping mode was used to determine the real strain. The analyzed region was a rectangular area measuring 100 mm in length and 10 mm in width.

Stress and strain were calculated in accordance with the ISO 527-5 standard. The elastic modulus was defined as the ratio between the stress and strain increments in the strain range between 0.0005 and 0.0025. The specimens had a width of 15.4 ± 0.6 mm and a thickness of 1.36 ± 0.09 mm. To achieve statistical significance, eight specimens were tested.

After testing, specimens were studied by scanning electron microscopy (SEM) using a Philips XL 30 SEM (Koninklijke Philips N.V., Amsterdam, The Netherlands) to study the fracture surfaces.

The reduction in the elastic modulus due to imperfect orientation experienced by the material can be approximated by utilizing classical laminate theory (CLT) [34]. Equation (1) can be applied to determine the longitudinal elastic modulus of a lamina *E_x_(α)* at a specific angle *α*.

(1) $$\frac{1}{E_{x}\left(\alpha \right)}={cos}^{4}\alpha \cdot \frac{1}{E_{1}}+{cos}^{2}\alpha \cdot {sin}^{2}\alpha \cdot \left(\frac{1}{G_{12}}-\frac{2\cdot \nu_{12}}{E_{1}}\right)+{sin}^{4}\alpha \cdot \frac{1}{E_{2}}$$

where *E_1_* and *E_2_* are, respectively, the longitudinal and transverse elastic moduli of a discontinuous unidirectional fiber lamina, *G_12_* is the in-plane shear modulus of the composite, and ν_12_ is the longitudinal Poisson’s ratio of the composite. *E_1_* is calculated using the modified rule of mixtures, with the elastic modulus of the reinforcement affected by the fiber length efficiency factor for short fiber composites η, which can be estimated using the shear lag theory (Cox model [35]).

*ν_12_* can be obtained using a linear rule of mixtures, considering the fiber volume fraction of the composite and Poisson’s ratios of the matrix and reinforcement. It should be noted that ν_12_ does not depend on fiber length. *E_2_* and *G_12_* have negligible dependence on fiber length. Both can be estimated using Chamis formulas, showing a dependence on the fiber volume fraction of the composite [36].

#### 2.3.2. Flexural Testing

Three-point bending tests were conducted in accordance with ISO 14125 in an Instron 5567 (Instron, Norwood, MA, USA) universal testing machine equipped with a 1 kN load cell. A span of 85 mm and a deflection rate of 1 mm/min were used for all tests, achieving a span-to-thickness ratio slightly higher than 60. High span-to-thickness ratios are recommended for flexural modulus determinations since shear deformation can notably reduce the apparent flexural modulus of composites when tested at low span-to-thickness ratios [37]. Tests were carried out under controlled humidity and temperature conditions (23 °C and 50% RH). The specimens had a width of 14.9 ± 0.2 mm and a thickness of 1.37 ± 0.04 mm, with the thickness being slightly smaller than the standard recommendation. Ten specimens were tested.

The flexural modulus of elasticity (E_f_) is the ratio of flexural stress and flexural strain in a strain range of 0.002. The start point and end point of the strain range are 0.0005 and 0.0025, respectively. The flexural modulus of elasticity is then calculated by linear regression in the mentioned strain range.

#### 2.3.3. Impact Testing

Impact tests were performed according to the ISO 6603-2 standard on an Instron/CEAST 9350 (Instron, Norwood, MA, USA) falling weight impact tester. The specimens were cut to 140 × 140 mm^2^ using a guillotine and were clamped on a support ring with an inner diameter of 40 mm and an outer diameter of 60 mm. The thickness of the specimen set was 1.69 ± 0.09 mm, and eight specimens were tested to ensure statistical significance. A hemispherical striker had a diameter of 20 mm, and a 20 kN load cell in the tip was dropped from a height of 1 m, with the specimens clamped at a force of 2800 N. The specimens were impacted by a total mass of 10.41 kg, corresponding to 102.1 J and resulting in an impact velocity of 4.4 m/s. As intended, full penetration was achieved in every case. The striker was blown with air and lubricated before every impact. All tests were performed at room temperature (23 °C and 50% RH).

The total energy absorbed during the impact test is calculated by integrating the full force–displacement curve. The following values are reported: the maximum force; the puncture deflection, corresponding to the deflection reached when the force has fallen to 50% of its maximum value; the puncture energy, which is the energy absorbed until reaching the puncture deflection; and the total energy absorbed, consisting of the integral of the area under the complete curve, i.e., until the force first reaches zero.

#### 2.3.4. Microcomputed Tomography

Microcomputed tomography (micro-CT) was used to study the composite specimens employed for impact testing before and after impact. The specimens were scanned using a GE Phoenix Nanotom S (General Electric, Boston, MA, USA) microtomography system. The specimens were cut into a rectangular shape, with an area of 40 × 40 mm^2^ and a thickness of 1.69 ± 0.09 mm. A high-power nanofocus X-ray tube was used along with a molybdenum target. The applied voltage and current were set at 60 kV and 140 mA, respectively, for a power of 8.4 W. A Hamamatsu Typ C7942SK-25 (Hamamatsu Photonics, Hamamatsu, Shizuoka, Japan) was used as a detector with a field of view of 120 × 120 mm^2^ and a resolution of 2300 × 2300 (5 megapixels). A voxel size of 16 μm was chosen for all the specimens.

Three specimens were analyzed, one of them before impact and the other two after impact. The specimens were placed in a rotating holder, and radiographs were recorded over a complete revolution. The radiographs were spaced at an interval of 0.15°/step, yielding a total of 2400 2D images. After image acquisition, the GE Phoenix Datos|X (General Electric, Boston, MA, USA) software was used for reconstruction. Subsequently, the 3D images were analyzed using the Avizo software (2021.1 version, Thermo Fisher Scientific, Waltham, MA, USA).

### 2.4. Thermal and Thermomechanical Behavior

#### 2.4.1. Thermomechanical Behavior

The thermomechanical behavior of the composites was evaluated according to the ISO 6721-11 standard and using a DMA Q800 (TA Instruments, New Castle, DE, USA) dynamic mechanical analyzer. These experiments were conducted with a fixed amplitude of 40 μm at a frequency of 1 Hz, selecting the multifrequency strain mode and using a single-cantilever configuration. The distance between the fixed and the movable clamp was 17.76 mm, and the specimens had a width of 15.3 ± 0.9 mm width and a thickness of 1.28 ± 0.03 mm. A temperature sweep was conducted from −40 °C to 200 °C, with a heating rate of 3 °C/min, including an isothermal step lasting for 5 min after reaching the minimum temperature. Five samples were tested for statistical significance.

After calculating the stress and strain, the elastic and viscous responses are obtained. The elastic response is characterized by the storage modulus (E′), while the viscous response is characterized by the loss modulus (E′′). The phase lag between stress and strain, also called loss factor, damping factor, or tan δ, is a key parameter obtained as the quotient between the loss modulus and the storage modulus. Tan δ is a measure of the energy dissipated as heat per cycle of deformation relative to the energy stored in the composite, therefore being related to its damping capacity [38].

The glass transition temperature (T_g_) was determined according to ISO 6721-11, which defines it as the temperature (1) at the inflection point of storage modulus, (2) at the peak of the loss modulus curve, or (3) at the peak of the loss factor curve. The loss modulus peak, acquired at 1 Hz, closely correlates with the T_g_ values obtained by differential scanning calorimetry (DSC) at a heating rate of 20 °C/min [26]. Unlike the storage modulus or loss factor (tan δ) peaks, the loss modulus peak represents an intermediate point [39].

#### 2.4.2. Differential Scanning Calorimetry

The T_g_ was determined using a DSC 882e (Mettler Toledo GmbH, Greifensee, Switzerland) differential scanning calorimetry (DSC). Specimens of 14 ± 3 mg of the composite were deposited in 40 μL aluminum crucibles with a 50 µm hole in the lid and were tested from 0 to 250 °C at a rate of 20 °C/min. Two different specimens were used, and two scans were performed on each specimen: the first one was to eliminate any absorbed moisture and erase the thermal history, and the second scan was to measure the T_g_. The purge gas used was nitrogen, supplied at a rate of 50 mL/min. The T_g_ was determined as the midpoint of the transition from the baseline to the extrapolated tangent of the transition. STARe software (version 12.10, Mettler Toledo GmbH, Greifensee, Switzerland) was used for data analysis.

## 3. Results and Discussion

### 3.1. Mechanical Characterization

#### 3.1.1. Tensile Strength

**Figure 2:**
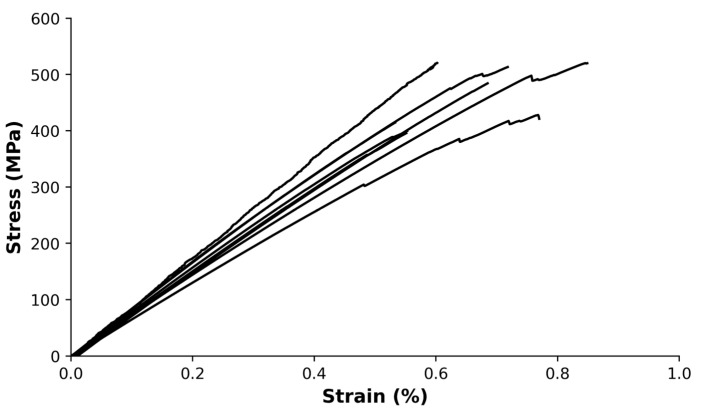
Stress–strain curves of the composites obtained through tensile tests.

The mechanical behavior of composites was evaluated through tensile, flexural, and impact tests. Figure 2 exhibits the stress–strain curves for the composites obtained in tensile testing. The curves indicate a brittle behavior, characterized by a linear region followed by a slightly noticeable nonlinear region until failure.

All tensile-tested specimens depicted in Figure 2 exhibited fractures that occurred at a significant distance from the grips, indicating that the grip pressure was appropriate and that the failure point can be relied on. The composite materials present an elastic modulus of 80 ± 14 GPa, an ultimate tensile strength of 460 ± 60 MPa, and a strain at break of 0.64 ± 0.14%. The coefficient of variation (CoV), calculated as the ratio of the standard deviation to the mean, was found to be 12%, 22%, and 17% for ultimate tensile strength, strain at break, and elastic modulus, respectively.

In a previous study [26], the authors utilized rods of mechanically recycled CFRP as reinforcement within a PA11 matrix, the composites being manufactured with the same process described above. In the present work, the mechanical properties of the composites were significantly improved by the inclusion of mechanically recycled aerospace prepreg waste. This is shown by an increase in ultimate tensile strength by 330% and elastic modulus by 275%, with a reduction in the strain at the break by 30%, along with lower measurement variability. Among the causes is the difference in the capillarity of the reinforcements. The rods utilized by Butenegro et al. [26] were quite compact, and their epoxy matrix was already completely cured prior to impregnation with PA11. As a result, the polyamide could not flow between the rods, resulting in a reduced contact surface between the reinforcement and the fibers. This was precisely one of the main motivations for using prepreg waste as reinforcement since the polyamide could more easily penetrate between them, significantly improving the reinforcement-matrix adhesion. Additionally, the main advantage of prepregs over rods lies in their superior aspect ratio, which allows the prepregs to build up stresses quicker.

The elastic modulus is directly related to the fiber content of the reinforcement and the misalignment of the reinforcement. Using the rule of mixtures, the volume of reinforcement present in the composites was calculated, obtaining a value of 51.5 ± 0.7%. By modeling the reinforcement as a laminate and utilizing CLT, the misalignment of the reinforcement effect can be considered. The misalignment angle ranged from 0 to 3°, both in the positive and negative sides of the longitudinal directions. The predicted elastic modulus by CLT ranged from 72 to 81 GPa, which is in line with the experimental results.

**Figure 3:**
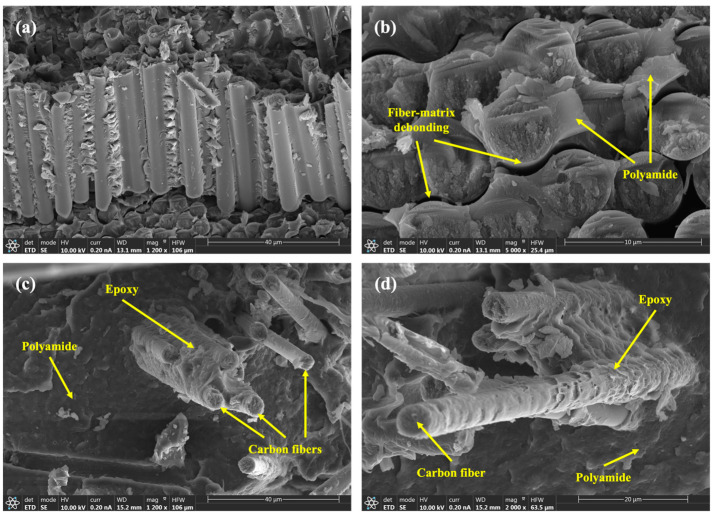
SEM micrographs corresponding to details of the fracture surface of composites that were subjected to tensile tests at different levels of magnification: (**a**) fracture surface at ×1200, (**b**) fiber-matrix debonding at ×5000, (**c**) fiber bundles covered in epoxy at ×1200, (**d**) fiber bundles coated in epoxy at ×2000.

Figure 3 presents a series of micrographs obtained through SEM, showcasing the fracture surfaces of the composites that were subjected to tensile tests.

Figure 3a illustrates the fracture surface resulting from the tensile tests, observed at a magnification of 1200. This micrograph reveals a bundle of fibers interconnected by the epoxy matrix, revealing fractured fibers. Figure 3b, captured at a magnification of 5000, exhibits the occurrence of fiber-matrix debonding following the edge of the fibers. Additionally, this micrograph reveals the brittle fracture of fibers, as seen in their cross-sections, and highlights the deformation of the surrounding polyamide, visible in a lighter tone. Figure 3c,d present detailed views of fibers observed at magnifications of 1200 and 2000, respectively. These micrographs also showcase the presence of epoxy-coated fiber bundles. Moreover, a good adhesion between the polyamide and the epoxy is observed. This good adhesion is mainly due to the chemical compatibility between the PA11 and the epoxy matrix. Additionally, epoxy and PA11 exhibit matching thermal expansion coefficients, which reduces the potential for delamination and improves the performance of the composites when subjected to varying temperatures. Notably, Figure 3c showcases the distinct imprint left on the polyamide matrix as a result of the pulled-off fibers.

#### 3.1.2. Flexural Strength

**Figure 4:**
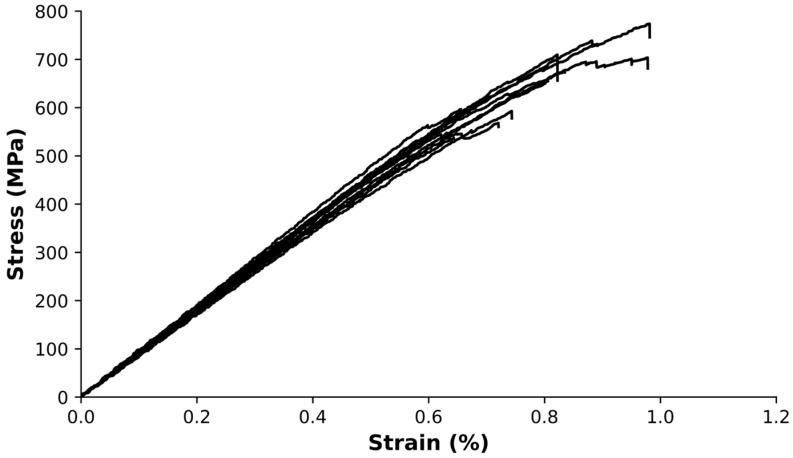
Stress–strain curves of the composites obtained through flexural tests.

Figure 4 displays the stress–strain curves obtained from the three-point bending tests, with each curve representing the behavior of a single specimen.

The composite material displayed a flexural modulus of 91 ± 3 GPa, a maximum stress of 650 ± 80 MPa, and a strain at maximum stress of 0.81 ± 0.12%. The coefficients of variation for these measurements were 12%, 4%, and 15%, respectively. It is worth noting that the high coefficient of variation values observed for maximum stress and strain at maximum stress is consistent with the anticipated variability in the mechanical performance of the composite.

The stacking sequence of reinforcement, matrix, and reinforcement layers during the manufacturing process resulted in a higher flexural modulus of elasticity compared to the tensile modulus of elasticity. In the three-point bending test, the composite experiences compression and tension on the top and bottom faces, respectively, while the middle section, where the neutral fiber is located, experiences minimal stress. The increased concentration of reinforcement near the top and bottom faces led to a local reinforcement volume exceeding the overall 51.5% volume fraction, resulting in a flexural modulus of elasticity value closer to the theoretical value, estimated to be around 92 GPa.

#### 3.1.3. Impact Resistance

Impact tests were performed to evaluate the response of the developed composite materials to an impact by a mass of 10.4 kg from a height of 1 m, i.e., with an impact energy of 102.1 J.

**Figure 5:**
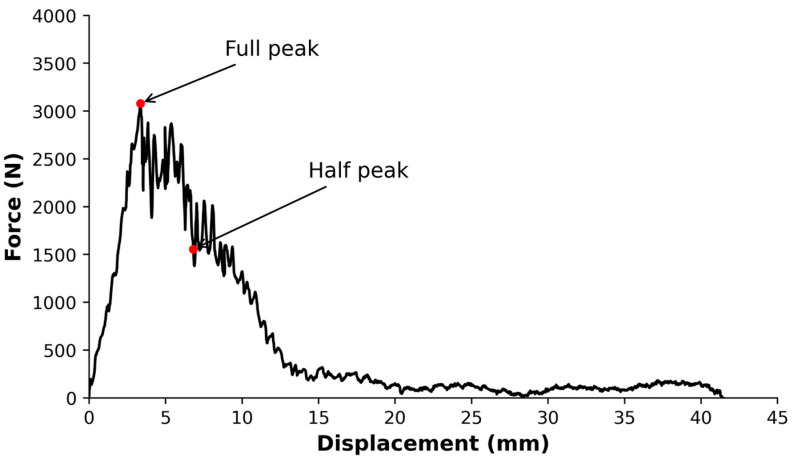
Representative force–displacement diagram for the penetration impact tests on the composites.

Figure 5 shows the force–displacement curve obtained during the impact testing of a representative specimen.

The slope of the linear region represents the stiffness of the specimens, while the area under the whole curve in Figure 5 represents the total energy absorbed by the specimens. The maximum force reached during the test was 3400 ± 700 N at a displacement of 3.5 ± 0.4 mm. The energy absorbed at peak load was 5.4 ± 1.8 J. The puncture deflection of the specimen was 6.9 ± 2.0 mm, and the corresponding puncture energy was 13.2 ± 3.0 J. The total energy absorbed by the specimen was 24.3 ± 2.2 J.

**Figure 6:**
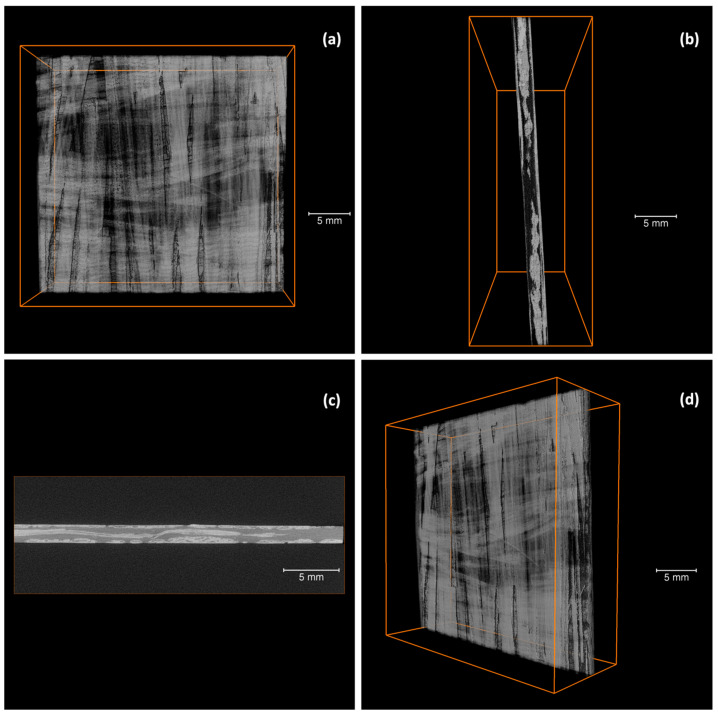
Micro-CT images of the manufactured composites before impact: (**a**) frontal view, (**b**) side view, (**c**) side view, (**d**) oblique view.

**Figure 7:**
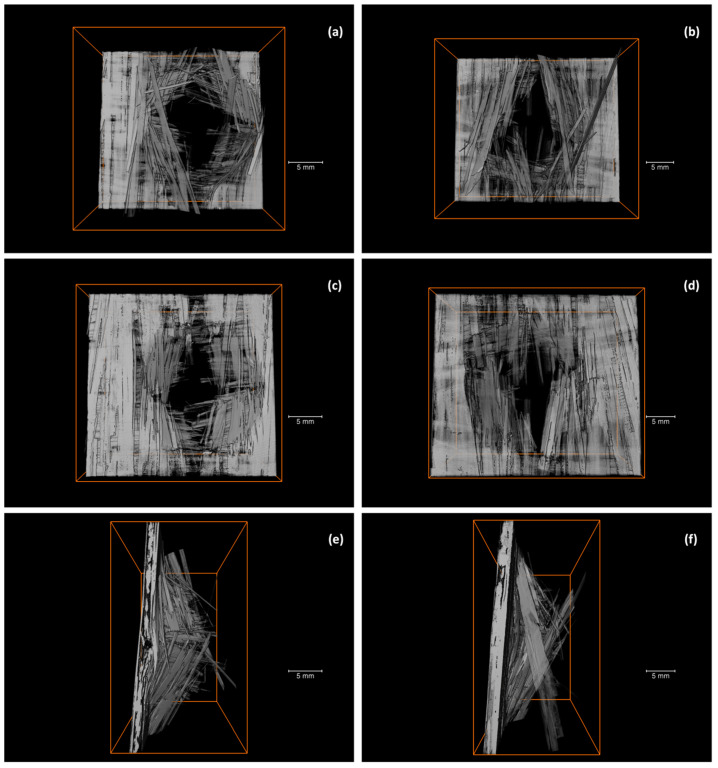
Micro-CT images of the manufactured composites after impact: (**a**) top view, (**b**) top view, (**c**) bottom view, (**d**) bottom view, (**e**) side view, (**f**) side view.

To understand the damage mechanisms that occur in the composites, three specimens were subjected to micro-CT scans. Figure 6 displays several reconstructed micro-CT pictures of specimens before impact, while Figure 7 exhibits the corresponding images of specimens after impact.

Figure 6 shows a section of the composite before impact. The two 0/90 directions are easily recognizable. In addition, it seems that the prepregs are relatively flexible. As the prepreg is not cured yet prior to processing, they can easily deviate out of the plane to accommodate prepregs in the other direction (see Figure 6c). However, residual stresses may remain in the material after cooling, potentially attributed to nonuniform cooling rates, differential shrinkage due to mismatched coefficients of thermal expansion, and fiber-matrix interactions involving differential thermal contraction [40,41].

Figure 7 exhibits two different types of impact damage patterns. Typically, the ply orientations are recognizable after impact. In the case of discontinuous fibers, in which each layer of fibers is oriented in the same direction, it can be understood as a ply, and the effect is similar. In Figure 7, although some fibers may appear to have broken, in general, the prepreg is observed to be pulling out of the matrix. This would explain the multiple jumps found in the drop in impact response in Figure 5. This is due to the length of the prepreg, which is not continuous over the entire surface. In Figure 7, the characteristic diamond shape resulting from the impact in a symmetrical 0/90/90/0 configuration can be noted, as well as the prepreg push-out, with some of the fibers fractured.

### 3.2. Thermomechanical Behavior

**Figure 8:**
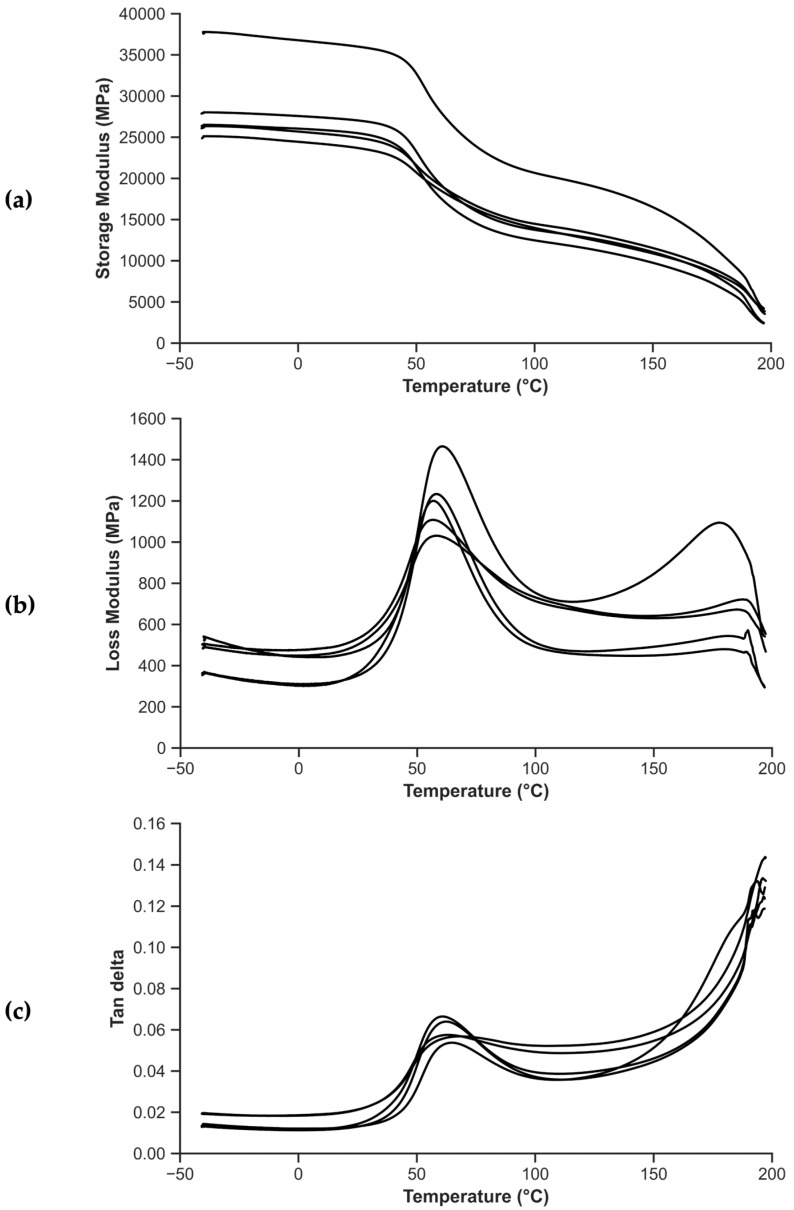
Thermomechanical behavior of composites as obtained after performing DMA tests: (**a**) storage modulus as a function of temperature, (**b**) loss modulus as a function of temperature, and (**c**) tan delta as a function of temperature.

Figure 8 presents the thermomechanical behavior of the composites as obtained after performing DMA tests. Figure 8a,b display the storage modulus and loss modulus as a function of temperature, respectively, while Figure 8c depicts the loss factor, or tan δ, as a function of temperature.

In Figure 8, the different behavior of the composites can be observed as a function of temperature. At temperatures below the T_g_, the material is in a glassy state, and the storage modulus, E′, is much higher than the loss modulus, E′′. This means that the material is relatively stiff and brittle, and it can store a large amount of elastic energy without much deformation. The tan δ is also relatively low in this region, indicating that the material has low damping capacity and is not very effective at dissipating mechanical energy. As the temperature is increased, the material undergoes a transition from a glassy state to a rubbery state, where E′ decreases rapidly and E′′ increases, indicating that the material becomes more viscous. The tan δ also increases in this region, indicating that the material has a higher damping capacity and is more effective at dissipating mechanical energy. The tan δ reaches a peak at the T_g_, indicating the maximum damping capacity of the material in this region.

Figure 8a includes a curve with a higher modulus than the rest, clearly displaying the material’s heterogeneity. A statistical study (Grubbs, α = 95%) indicates that the corresponding specimen cannot be discarded. The tan δ peak in Figure 8c has a height that is lower than one, which suggests that there is no nucleation happening between the epoxy and polyamide in the prepreg.

**Figure 9:**
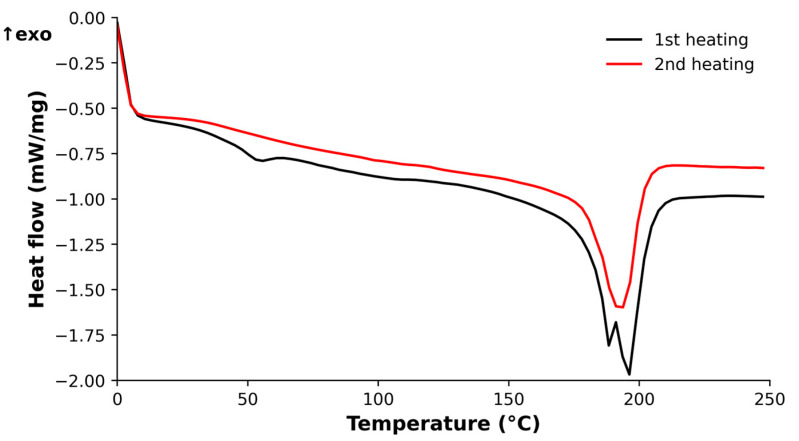
DSC thermograms of the prepreg-reinforced manufactured composites.

From Figure 8, T_g_ values are obtained, depending on whether the onset in the storage modulus, the peak of the loss modulus, or the peak of the tan δ is considered, respectively. Figure 9 shows the DSC thermograms of the prepreg-reinforced manufactured composites, which allow the calculation of T_g_. The vertical axis represents the heat flow normalized by the weight of the specimen.

As is common in DSC analysis, the T_g_ is more clearly observed in the first heating. Around 180 °C, two overlapping peaks are observed. One corresponds to the T_m_ of PA11, while the other reflects the reported T_g_ of the prepreg, with a high enthalpy of relaxation. The equivalent of these peaks can be seen in some curves of Figure 8, especially when examining the loss modulus (see Figure 8b) or the tan δ (see Figure 8c). Compared with a study carried out by Butenegro et al. [26], the T_g_ of the polyamide in the case of composites remains unchanged (51.1 ± 1.6 °C in the present study compared to 52.8 ± 1.0 °C in the mentioned study). Table 1 compares the T_g_ values obtained by DSC and DMA with those obtained in previous studies. At a temperature of 180 °C, the T_g_ of the epoxy and the T_m_ of the polyamide occur simultaneously.

Bahrami et al. [17] obtained the T_g_ of the polyamide using DSC at a heating rate of 20 °C/min. The considered T_g_ value corresponds to the second heating cycle after erasing PA11 thermal history [42]. Butenegro et al. [26] obtained the T_g_ of the polyamide and that of composites manufactured with reused CFRP and polyamide using DMA at a heating rate of 3 °C/min. Due to the addition of rods or prepregs as reinforcement, an overall increase in T_g_ is observed in the composites, both for DSC and DMA. DSC analysis showed an increase of approximately 9 °C in the T_g_ during the second heating cycle. In DMA, the differences in T_g_ between PA11 and the composite were more notable when analyzing tan δ and less pronounced when studying E′. E′′ constituted an intermediate case between the two.

The values of storage modulus, loss modulus, and tan δ at three different temperatures are reported in Table 2 for the prepreg-reinforced composites manufactured, as well as the CoV obtained.

The temperatures chosen in Table 2 are (1) at room temperature or 25 °C, (2) at the T_g_ of the polyamide, and (3) at 100 °C, a temperature much higher than the polyamide T_g_. In line with the rest of the tests, the heterogeneity of the material is well represented by the CoV. This is further highlighted by the observations made regarding Figure 8a, where one of the curves significantly deviates from the others until the melting temperature of PA11 is reached. Nevertheless, the storage modulus at room temperature, with the material still in a glassy state, is 95% of its maximum value. Around T_g_, the storage modulus value drops to 80% of its maximum value. Finally, at 100 °C, in a zone comparable to a rubber elastic plateau, the storage modulus drops to about 50% of its maximum value. Above 100 °C, the mobility of the polymer chains increases, leading to more extensive chain movements and reduced elastic behavior. This results in the observed decrease in the storage modulus, indicating the transition from the rubbery plateau to a more viscous behavior.

## 4. Conclusions

In this research paper, we developed and carried out a mechanical, thermal, and thermomechanical characterization of a composite consisting of a PA11 matrix reinforced with mechanically recycled aerospace prepreg. The following conclusions can be drawn:

- The manufacturing process for a new thermoplastic composite, incorporating mechanically recycled aerospace prepreg as reinforcement, has been effectively implemented, enabling the study of its properties.
- The mechanical tests exhibited the inherent heterogeneity of the recycled composites, reflected in the large coefficients of variation. The longitudinal elastic modulus of the composites is influenced by the fiber content and misalignment of the reinforcement. The volume fraction of reinforcement was determined to be approximately 51.5%, and the misalignment angle ranged from 0 to 3°. The predicted elastic modulus in tension using CLT was in the range of 72 to 81 GPa, consistent with experimental results. Regarding flexural strength tests, the stacking sequence during manufacturing led to a higher flexural modulus compared to the tensile modulus.
- Impact penetration tests, aided by micro-CT images, have allowed for the study of composite behavior under impact. The composite absorbs nearly 25% of the energy until reaching maximum force and over 50% of the total energy during puncture deflection when the force first drops to 50% of the maximum. Micro-CT images revealed the characteristic diamond shape seen in woven composites and perpendicularly oriented layered composites. While some fibers may exhibit signs of breakage, the overall observation indicates that the prepregs tend to pull out from the matrix, providing a potential explanation for the multiple drops in impact strength.
- The T_g_ values obtained through DSC and DMA are similar, being 54.7 °C and 51.1 ± 1.6 °C, respectively. In the case of DMA, it is observed that the T_g_ increases for the composites, which could be attributed to the influence of the fibers on the crystallinity of PA11. The cooling rate during the composite manufacturing process may also have an impact in the same direction.

Our future research aims to explore the recycling and reutilization of end-of-life or expired aeronautical and automotive composites while preserving their original matrices. This includes their incorporation into biobased polyamide to develop sustainable materials, as well as the use of liquid thermoplastic matrices. Additionally, the application of these composites to reinforce vehicle structures to improve their impact resistance is being investigated.

## Figures and Tables

**Table 1:** Glass transition temperature by technique for PA11 and composites.

Material	Glass Transition Temperature (°C) by Technique				Ref.
	DSC	DMA			
		E′	E′′	Tan δ	
PA11 (DSC)	46.0	-	-	-	[17]
PA11 (DMA)	-	47.5 ± 0.4	49.9 ± 0.4	51.0 ± 1.7	[26]
Rod-reinforced composite	-	52.8 ± 1.0	55.7 ± 1.1	-	[26]
Prepreg-reinforced composite	54.7	51.1 ± 1.6	58.1 ± 1.6	63.9 ± 2.9	Present study

**Table 2:** Storage modulus, loss modulus, and tan δ for the prepreg-reinforced composites at three different temperatures.

Material	Temperature (°C)	Storage Modulus (MPa) [CoV]	Loss Modulus (MPa) [CoV]	Tan δ [CoV]
Prepreg-reinforced composites	25 °C	27,400 ± 4900 [18%]	440 ± 80 [18%]	0.017 ± 0.004 [26%]
	T_g_ = 51 ± 2 °C	23,000 ± 4800 [21%]	1100 ± 80 [7%]	0.049 ± 0.007 [13%]
	100 °C	15,100 ± 3200 [21%]	640 ± 130 [20%]	0.043 ± 0.007 [17%]

## Data Availability

Not applicable.

## References

[1] Zhang J., Lin G., Vaidya U., Wang H. (2023). Past, Present and Future Prospective of Global Carbon Fibre Composite Developments and Applications. Compos. Part B Eng..

[2] Kupski J., Teixeira de Freitas S. (2021). Design of Adhesively Bonded Lap Joints with Laminated CFRP Adherends: Review, Challenges and New Opportunities for Aerospace Structures. Compos. Struct..

[3] Al-Lami A., Hilmer P., Sinapius M. (2018). Eco-Efficiency Assessment of Manufacturing Carbon Fiber Reinforced Polymers (CFRP) in Aerospace Industry. Aerosp. Sci. Technol..

[4] Galvez P., Quesada A., Martinez M.A., Abenojar J., Boada M.J.L., Diaz V. (2017). Study of the Behaviour of Adhesive Joints of Steel with CFRP for Its Application in Bus Structures. Compos. Part B Eng..

[5] Lavayen-Farfan D., Butenegro-Garcia J.A., Boada M.J.L., Martinez-Casanova M.A., Rodriguez-Hernandez J. (2022). Theoretical and experimental study of the bending collapse of partially reinforced CFRP-Steel square tubes. Thin-Walled Struct..

[6] Chen C.-H., Chiang C.-L., Wang J.-X., Shen M.-Y. (2022). A Circular Economy Study on the Characterization and Thermal Properties of Thermoplastic Composite Created Using Regenerated Carbon Fiber Recycled from Waste Thermoset CFRP Bicycle Part as Reinforcement. Compos. Sci. Technol..

[7] Rubino F., Nisticò A., Tucci F., Carlone P. (2020). Marine Application of Fiber Reinforced Composites: A Review. J. Mar. Sci. Eng..

[8] Xiong Z., Wei W., Liu F., Cui C., Li L., Zou R., Zeng Y. (2021). Bond Behaviour of Recycled Aggregate Concrete with Basalt Fibre-Reinforced Polymer Bars. Compos. Struct..

[9] Akbar A., Liew K.M. (2020). Assessing Recycling Potential of Carbon Fiber Reinforced Plastic Waste in Production of Eco-Efficient Cement-Based Materials. J. Clean. Prod..

[10] Butenegro J.A., Bahrami M., Abenojar J., Martínez M.Á. (2021). Recent Progress in Carbon Fiber Reinforced Polymers Recycling: A Review of Recycling Methods and Reuse of Carbon Fibers. Materials.

[11] Stieven Montagna L., Ferreira de Melo Morgado G., Lemes A.P., Roberto Passador F., Cerqueira Rezende M. (2022). Recycling of Carbon Fiber-Reinforced Thermoplastic and Thermoset Composites: A Review. J. Thermoplast. Compos. Mater..

[12] Sajan S., Philip Selvaraj D. (2021). A Review on Polymer Matrix Composite Materials and Their Applications. Mater. Today Proc..

[13] Alshammari B.A., Alsuhybani M.S., Almushaikeh A.M., Alotaibi B.M., Alenad A.M., Alqahtani N.B., Alharbi A.G. (2021). Comprehensive Review of the Properties and Modifications of Carbon Fiber-Reinforced Thermoplastic Composites. Polymers.

[14] Hegde S., Satish Shenoy B., Chethan K.N. (2019). Review on Carbon Fiber Reinforced Polymer (CFRP) and Their Mechanical Performance. Mater. Today Proc..

[15] Park M., Hong S.-J., Lee S., Kim N.-K., Shin J., Kim Y.-W. (2022). Effects of Hard Segment Length on the Mechanical Properties of Poly(PA11-*Co*-DA) Periodic Copolymers. ACS Sustain. Chem. Eng..

[16] Feldmann M., Bledzki A.K. (2014). Bio-Based Polyamides Reinforced with Cellulosic Fibres—Processing and Properties. Compos. Sci. Technol..

[17] Bahrami M., Abenojar J., Martínez M.A. (2021). Comparative Characterization of Hot-Pressed Polyamide 11 and 12: Mechanical, Thermal and Durability Properties. Polymers.

[18] Zhang J., Chevali V.S., Wang H., Wang C.-H. (2020). Current Status of Carbon Fibre and Carbon Fibre Composites Recycling. Compos. Part B Eng..

[19] Wang Y., Li A., Zhang S., Guo B., Niu D. (2023). A Review on New Methods of Recycling Waste Carbon Fiber and Its Application in Construction and Industry. Constr. Build. Mater..

[20] Gopalraj S.K., Kärki T. (2020). A Review on the Recycling of Waste Carbon Fibre/Glass Fibre-Reinforced Composites: Fibre Recovery, Properties and Life-Cycle Analysis. SN Appl. Sci..

[21] Naqvi S.R., Prabhakara H.M., Bramer E.A., Dierkes W., Akkerman R., Brem G. (2018). A Critical Review on Recycling of End-of-Life Carbon Fibre/Glass Fibre Reinforced Composites Waste Using Pyrolysis towards a Circular Economy. Resour. Conserv. Recycl..

[22] Gopalraj S.K., Kärki T. (2020). A Study to Investigate the Mechanical Properties of Recycled Carbon Fibre/Glass Fibre-Reinforced Epoxy Composites Using a Novel Thermal Recycling Process. Processes.

[23] Jiang J., Deng G., Chen X., Gao X., Guo Q., Xu C., Zhou L. (2017). On the Successful Chemical Recycling of Carbon Fiber/Epoxy Resin Composites under the Mild Condition. Compos. Sci. Technol..

[24] Khalil Y.F. (2019). Sustainability Assessment of Solvolysis Using Supercritical Fluids for Carbon Fiber Reinforced Polymers Waste Management. Sustain. Prod. Consum..

[25] Butenegro J.A., Bahrami M., Martínez M.Á., Abenojar J. (2023). Reuse of Carbon Fibers and a Mechanically Recycled CFRP as Rod-like Fillers for New Composites: Optimization and Process Development. Processes.

[26] Butenegro J.A., Bahrami M., Swolfs Y., Ivens J., Martínez M.Á., Abenojar J. (2022). Novel Thermoplastic Composites Strengthened with Carbon Fiber-Reinforced Epoxy Composite Waste Rods: Development and Characterization. Polymers.

[27] Wan Y., Takahashi J. (2016). Tensile and Compressive Properties of Chopped Carbon Fiber Tapes Reinforced Thermoplastics with Different Fiber Lengths and Molding Pressures. Compos. Part A Appl. Sci. Manuf..

[28] Palmieri B., Borriello C., Rametta G., Iovane P., Portofino S., Tammaro L., Galvagno S., Giordano M., Ambrosio L., Martone A. (2023). Investigation on Stress Relaxation of Discontinuous Recycled Carbon Fiber Composites. J. Mater. Eng. Perform..

[29] Gillet A., Mantaux O., Cazaurang G. (2015). Characterization of Composite Materials Made from Discontinuous Carbon Fibres within the Framework of Composite Recycling. Compos. Part A Appl. Sci. Manuf..

[30] Barnett P.R., Vigna L., Martínez-Collado J.L., Calzolari A., Penumadu D. (2023). Crashworthiness of Recycled Carbon Fiber Composite Sinusoidal Structures at Dynamic Rates. Compos. Struct..

[31] Stelzer P.S., Cakmak U., Eisner L., Doppelbauer L.K., Kállai I., Schweizer G., Prammer H.K., Major Z. (2022). Experimental Feasibility and Environmental Impacts of Compression Molded Discontinuous Carbon Fiber Composites with Opportunities for Circular Economy. Compos. Part B Eng..

[32] Boisot G., Fond C., Hochstetter G., Laiarinandrasana L. (2008). Failure of Polyamide 11 Using a Damage Finite Elements Model. Proceedings of the 17th European Conference on Fracture.

[33] García-Moreno I., Caminero M., Rodríguez G., López-Cela J. (2019). Effect of Thermal Ageing on the Impact and Flexural Damage Behaviour of Carbon Fibre-Reinforced Epoxy Laminates. Polymers.

[34] Tang J., Swolfs Y., Longana M.L., Yu H., Wisnom M.R., Lomov S.V., Gorbatikh L. (2019). Hybrid Composites of Aligned Discontinuous Carbon Fibers and Self-Reinforced Polypropylene under Tensile Loading. Compos. Part A Appl. Sci. Manuf..

[35] Cox H.L. (1952). The Elasticity and Strength of Paper and Other Fibrous Materials. Br. J. Appl. Phys..

[36] Chamis C.C. (1989). Mechanics of Composite Materials: Past, Present, and Future. J. Compos. Technol. Res..

[37] Zhang Y., Meng L., Wan Y., Xiao B., Takahashi J. (2021). Measurement of the Flexural Modulus of Chopped Carbon Fiber Tape Reinforced Thermoplastic with Short Beams. Appl. Compos Mater..

[38] Abenojar J., Tutor J., Ballesteros Y., del Real J.C., Martínez M.A. (2017). Erosion-Wear, Mechanical and Thermal Properties of Silica Filled Epoxy Nanocomposites. Compos. Part B Eng..

[39] Ribeiro F., Sena-Cruz J., Vassilopoulos A.P. (2021). Tension-Tension Fatigue Behavior of Hybrid Glass/Carbon and Carbon/Carbon Composites. Int. J. Fatigue.

[40] Xia H., Ma Y., Chen C., Su J., Zhang C., Tan C., Li L., Geng P., Ma N. (2022). Influence of Laser Welding Power on Steel/CFRP Lap Joint Fracture Behaviors. Compos. Struct..

[41] Rohrmüller B., Gumbsch P., Hohe J. (2021). Calibrating a Fiber–Matrix Interface Failure Model to Single Fiber Push-out Tests and Numerical Simulations. Compos. Part A Appl. Sci. Manuf..

[42] Abenojar J., Lopez de Armentia S., Barbosa A.Q., Martinez M.A., del Real J.C., da Silva L.F.M., Velasco F. (2023). Magnetic Cork Particles as Reinforcement in an Epoxy Resin: Effect of Size and Amount on Thermal Properties. J. Therm. Anal. Calorim..

